# Evidence for Persistent Monocyte and Immune Dysregulation After Prolonged Viral Suppression Despite Normalization of Monocyte Subsets, sCD14 and sCD163 in HIV-Infected Individuals

**DOI:** 10.20411/pai.v4i2.336

**Published:** 2019-12-17

**Authors:** Anjana Yadav, Andrew V. Kossenkov, Vincent R. Knecht, Louise C. Showe, Sarah J. Ratcliffe, Luis J. Montaner, Pablo Tebas, Ronald G. Collman

**Affiliations:** 1 Department of Medicine; University of Pennsylvania Perelman School of Medicine; Philadelphia, Pennsylvania; 2 Department of Biostatistics and Epidemiology; University of Pennsylvania Perelman School of Medicine; Philadelphia, Pennsylvania; 3 The Wistar Institute; Philadelphia, Pennsylvania

**Keywords:** HIV/AIDS, Antiretroviral therapy, Monocyte, Gene expression, Cytokine, Inflammation

## Abstract

**Background:**

People living with HIV on antiretroviral therapy (HIV/ART) experience excess non-AIDS comorbidities, and also remain at increased risk for certain infections and viral malignancies. Monocytes/macrophages are central to many of these comorbidities, and elevated plasma cytokines and immune activation during untreated infection are often incompletely reversed by ART and are also associated with comorbidities.

**Methods:**

We investigated monocyte surface markers, gene expression, and plasma cytokines in 11 HIV-infected older individuals (median 53 years) who started therapy with low CD4 counts (median 129 cells/µl), with elevated hsCRP (≥ 2mg/L) despite long-term ART (median 7.4 years), along with matched controls.

**Results:**

Frequency of monocyte subsets (based on CD14/CD16/CD163), were not different from controls, but surface expression of CD163 was increased (*P* = 0.021) while PD1 was decreased (*P* = 0.013) along with a trend for higher tissue factor (*P* = 0.096). As a group, HIV/ART participants had elevated plasma CCL2 (MCP-1; *P* = 0.0001), CXCL9 (MIG; *P* = 0.04), and sIL2R (*P* = 0.015), which were correlated, while sCD14 was not elevated. Principal component analysis of soluble markers revealed that 6/11 HIV/ART participants clustered with controls, while 5 formed a distinct group, driven by IL-10, CCL11, CXCL10, CCL2, CXCL9, and sIL2R. These individuals were significantly older than those clustering with controls. Transcriptomic analysis revealed multiple genes linked to immune functions including inflammation, immune cell development, and cell-cell signaling that were downregulated in HIV/ART monocytes and distinct from patterns in untreated subjects.

**Conclusions:**

Long-term ART-treated individuals normalize monocyte subsets but exhibit immune dysregulation involving both aberrant inflammation and monocyte dysfunction, as well as inter-individual heterogeneity, suggesting complex mechanisms linking monocytes and HIV/ART comorbidities.

## INTRODUCTION

Antiretroviral therapy (ART) can suppress virus replication, extend lifespan, and improve quality of life for people living with HIV but does not completely eliminate chronic immune activation and inflammation [1, 2]. The principal causes of morbidity and death for people with HIV in the US has now shifted from AIDS-related opportunistic infections to chronic diseases and non-AIDS complications, including HIV-associated neurocognitive disorders (HAND), cardiovascular disease (CVD), non-AIDS cancers, and bone and metabolic disorders, among others. These serious non-AIDS events (SNAEs) in HIV/ART individuals are associated with, and are believed to be driven in large part by persistent chronic inflammation [3]. At the same time, these individuals remain at elevated risk for certain infections such as tuberculosis and for virally-induced cancers [4–6].

Monocytes and macrophages are key components of chronic inflammation in ART-treated HIV infection, and both cellular and soluble myeloid-associated markers have been linked to development of HAND, CVD, and other SNAEs [7–10]. In HAND, persistent neuroinflammation is linked to the accumulation of activated macrophages in the CNS, which results from emigration of monocytes trafficking from the blood [11, 12]. This mechanism is supported by elevated levels of IL-6, IL-8, IFN-γ, and sCD14 in plasma, and of CCL2 (MCP-1) and CXCL10 in the CNS. The latter chemokines have been shown to be involved in monocyte chemotaxis in ART-treated HIV infected individuals with HAND [13, 14]. Similarly, a monocyte/macrophage role in CVD is supported by biomarker data in ART-treated individuals, including elevated sCD14, CCL2, IL-6, and other plasma biomarkers [13, 15–17]. The impact of ART treatment on monocyte CD16+ populations is less clear, with some studies reporting persistent elevated expression, but others reporting normalization [18–22]. Importantly, both residual inflammation and comorbidities such as HAND are most common in those who begin ART at low CD4+ T counts and in older individuals [23, 24]

To better understand the nature of residual monocyte dysfunction and its relationship to persistent immune activation in ART-suppressed individuals, we investigated monocyte surface marker phenotypes, monocyte gene expression patterns, and plasma biomarkers in HIV/ART subjects. We selected aviremic HIV+ individuals without clinical neurocognitive, cardiovascular, or inflammatory diseases, but with elevated hsCRP (≥ 2 mg/L) as a marker of inflammation despite long-term ART treatment (median 7.4 years). To study the group most at risk for SNAEs, we recruited subjects who began ART with advanced disease (median CD4 of 129 cells/µl at time of ART initiation) and a median age of 53. Healthy controls were matched for age, race, gender, and smoking status. Results of this study highlight the continued dysregulation of myeloid cells, and have implication for immune activation, inflammation, and suboptimal recovery of immune function.

## METHODS

### Patient Recruitment and Blood Collection

HIV+ ART-treated participants (Table 1) were recruited based on nadir CD4 count ≤350 cells/µl, HIV-1 RNA ≤200 copies/ml for ≥ 6 months, and plasma hsCRP ≥ 2mg/L. Participants were required to be on a stable ART regimen for at least 4 weeks before enrollment, and were excluded based on any known inflammatory conditions, hepatitis C infection, clinical cardiovascular or coronary artery disease, hyperlipidemia or use of statin drugs, use of any prescription anti-inflammatory drugs, or non-steroidal anti-inflammatory drug use on a regular basis. Healthy HIV-negative controls with the same exclusion criteria were matched for race, gender, age (+/− 5 years), and smoking status. All participants provided written informed consent under protocol #815512 approved by the University of Pennsylvania Institutional Review Board.

**Table 1.** Subjects enrolled in the study

PID^a^	Age	Race^b^	Sex	Smoker	hsCRP at entry (mg/L)	ART Regimen^b^	Time on ART (years)	CD4 nadir (cells/μl)	CD4 at entry (cells/μl)
**H117**	31	AA	M	no	8.8	ATV.RTV, ABC, 3TC	10	27	413
**H118**	45	C	M	no	5.4	ABC, 3TC, ATV	9	98	677
**H119**	36	AA	M	no	1.6 (2.1)^c^	EFV, FTC, TDF	5	155	342
**H120**	37	AA	M	no	2.7	DRV, RTV, TDF, FTC	6	166	510
**H121**	33	AA	F	no	5.4	RAL, TDF, FTC	7	233	1062
**H122**	53	AA	M	yes	2.9	EFV, FTC, TDF	7	129	172
**H123**	62	AA	F	no	9.7	EFV, FTC, TDF	7	182	503
**H124**	58	AA	M	no	2.5	LPV, RTV, 3TC, AZT	13	218	587
**H125**	62	AA	M	no	4	EFV, FTC, TDF	5	27	529
**H101**	60	AA	F	no	3.2	LPV, RTV, 3TC, AZT	6	24	914
**H102**	55	AA	M	yes	9.7	EVG, FTC, TDF, cobicistat	3	84	620
**C345**	30	AA	M	no	0.9				
**C121**	38	C	M	no	1.3	-	-	-	-
**C420**	38	AA	M	no	0.2	-	-	-	-
**C534**	29	AA	M	no	1.3	-	-	-	-
**C458**	33	AA	F	no	0.9	-	-	-	-
**C383**	56	AA	M	yes	5.1				
**C227**	60	AA	F	no	8.3	-	-	-	-
**C469**	51	AA	M	no	0.4				
**C30**	59	AA	M	no	1.6	-	-	-	-
**C550**	66	AA	F	no	2.3	-	-	-	-
**C188**	58	AA	M	yes	4.0	-	-	-	-

a HIV-controls (C) were matched to HIV+ subjects (H) by age, race, gender, and smoking status.

b Abbreviations: AA, African American; C, Caucasian; ATV, atazanavir; RTV, ritonavir; ABC, abacavir; 3TC, lamivudine; EFV, efavirenz; FTC, emtricitabine; TDF, tenofovir; DRV, darunavir; RAL, raltegravir; LPV, lopinavir; AZT, zidovudine; EVG, elvitegravir.

c hsCRP ≥ 2mg/L enrollment criterion was fulfilled by H119 at screening but was 1.6 at entry visit.

### Purification of CD14+ Monocytes

Blood was collected in EDTA tubes and processed within 3 hrs. Plasma was separated and stored at −80^o^C for ELISA and bead-based multiplex assays. PBMCs were separated by Ficoll-gradient centrifugation followed by purification of CD14+ monocytes by negative selection with antibody-conjugated magnetic beads according to the manufacturer's instructions (Miltenyi Biotech).

### Flow Cytometry

**Supplementary Figure 1.**
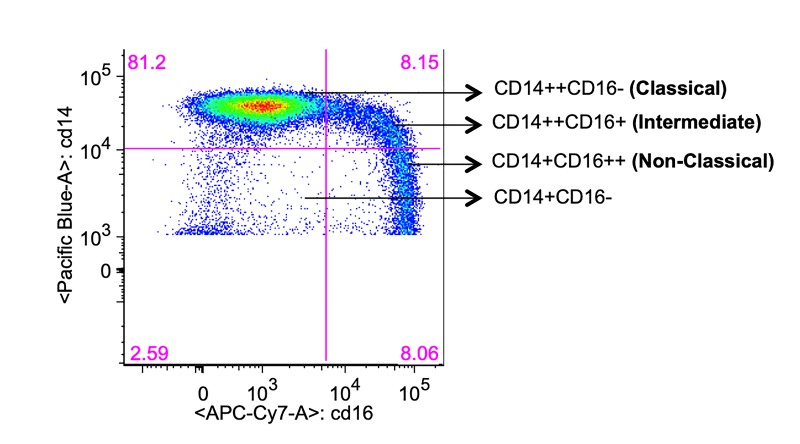
**Gating of monocyte subsets.** Freshly isolated PBMCs from HIV+ and HIV-individuals were stained for monocyte surface markers and analyzed by flow cytometry. Representative dot plot showing monocyte subsets based on CD16 expression after gating on total CD14+ monocytes.

For analysis of cell surface antigens, fresh whole blood was stained with a cocktail of antibodies that included CD14-Pacific Blue, CD16-Apc-Cy7, CD163-PerCpCy5-5, CD3-BV570, CD4-Pe-Cy5, CD8-PeTexRed, CX3CR1-FITC, CCR2-Pe, CD38-PeCy7, CD142-Pe, PD1-PeCy7, and PD-L1-APC. Briefly, the antibody cocktail was added to 100µl of whole blood, vortexed gently, and incubated in the dark at room temperature for 20 minutes. Next, 2ml of RBC lysing buffer was added, vortexed, and incubated for 10 minutes in the dark at room temperature. Cells were centrifuged at 500g for 5 minutes to remove the supernatant, washed twice in 2ml of FACS buffer, and suspended in 2% paraformaldehyde and stored at 4^o^C until acquisition. FACS data were acquired on a modified LSRII (BD Immunocytometry Systems) and analyzed using FlowJo (TreeS-tar) software. For each stain, an FMO (fluorescence-minus-one) tube was included as a control to establish gating. Gating strategy is shown in Supplementary Figure 1.

### Plasma Assays

Levels of sCD14 and sCD163 were measured in plasma by ELISA (R&D). Plasma LBP (LPS binding protein) was also measured by ELISA (Cell Sciences). All other cytokines and chemokines were measured using the Luminex multiplex human cytokine assay kit (catalog no: LHC0009, Invitrogen).

### RNA isolation and Microarray Assays

Total RNA was isolated from purified monocytes using the Qiagen DNA/RNA mini kit (catalog no. 80204). RNA quality was assayed by Eukaryote total RNA nano Bioanalyzer (Agilent) assay, and all RNA used had an RNA Integrity number (RIN) > 7. RNA (100ng) was amplified with Epicentre TargetAmp Nano-g Biotin-aRNA Labeling Kit to generate biotinylated amplified RNA. Biotin labeled aRNA (750ng) was hybridized to the Illumina HumanHT-12V4 expression Beadchip following the manufacturer's instructions. Illumina GenomeStudio software was used to export expression levels and determination of *P*-values for each probe of each sample. Signal intensity data was quantile normalized, and probes that showed non-significant detection *P*-value (*P* > 0.05) in all samples were removed from further analysis, resulting in a set of 29,208 probes (20,464 unique genes). Microarray data is available on GEO (https://www.ncbi.nlm.nih.gov/geo) using accession GSE137438.

Gene expression levels between the HIV/ART and control groups were compared using two sample SAM test [25]. FDR < 20% was used as a significance threshold for general enrichment analysis, and FDR < 10% with fold > 1.5 enrichment threshold was used to report the most significantly changed genes. Gene set enrichment analysis for biological functions and pathways was done using Ingenuity Pathway Analysis (IPA) software (Qiagen) using “Canonical Pathways” and “Disease and Functions” options. Results that passed the *P* < 0.01 threshold with significant predicted activation state (Z-score of at least 2) were reported. Database for Annotation Visualization and Integrated Discovery (DAVID) analysis was performed to find genes having known HIV interaction [26]. Additional enrichment analysis was done using GSEA [27] on genes pre-ranked by SAM significance estimation without using direction of change and using 1000 permutations to find significantly associated pathways (MSigDB set C2) with FDR < 25% used for significance threshold.

### Quantitative Real-Time PCR

Quantitative real-time PCR was carried out for selected genes from microarray data based on fold change. Monocyte RNA was purified using the RNeasy Plus Mini Kit (Qiagen) following the manufacturers' instructions. cDNA was prepared using the High-Capacity cDNA Reverse Transcription Kit (Applied Biosystems). Real-time PCR was carried out on an ABI 7500 Fast Real-Time PCR system using SYBR Select Master Mix (Applied Biosystems). Gene targets and primers used were: CD247 5'-TGCTGGATGGAATCCTCTTC-3' and 3'-CCGCCATCTTATCTTTCTGC-5'; IL2RB 5'-GCTGATCAACTGCAGGAACA-3' and 3'-TGTCCCTCTTCCAGCACTTCT-5'; KIR3DL1 5'-CAAGCTCCAAATCTGGTAACCC-3' and 3'-CCAACTGTGCGTATGTCACC-5'; KIR3DL2 5'-AGGGCCCCTGCTGAAATC-3' and 3'-GCTCAAACATGACATCTGACCAA-5'; and housekeeping gene targets: IPO8 5'-GCTCTGATAACTGTGCAG-3' and 3'-CAGTGTGTACACCTCCTG-5'; GAPDH 5'-GGTGGTCTCCTCTGACTTCAACA-3' and 3'-CCAGCCACATACCAGGAAATG-5'. Each PCR reaction was performed using 2.5µl of cDNA and forward and reverse primers each at 200nm final concentration, 20µl reaction volumes. No-template and no-RT controls were run in parallel for each gene and each sample. Cycling parameters were: 50^o^C for 2 minutes, 95^o^C for 10 minutes, then 40 cycles of 94^o^C for 30 seconds, 58^o^C for 30 seconds, and 72^o^C for 45 seconds, and one cycle at 72^o^C for 3 minutes. Melt curve analysis steps were 95^o^C for 15 seconds, 60^o^C for 20 seconds and 95^o^C for 15 seconds. Gene expression was calculated by the 2^−ΔC^_T_ method [28] based on relative expression compared to internal control IPO8.

### Statistical Analyses

Statistical evaluations of monocyte surface and plasma markers were carried out using the non-parametric-unpaired Mann-Whitney test, and a *P* value of < 0.05 was considered to be significant. Results are expressed as mean ± standard error of mean (SEM). Correlation analysis was carried out using the Spearman correlation test. Principal component analysis (PCA) of plasma cytokines and chemokines was carried out using R-Studio statistical software.

## RESULTS

### Participant Characteristics

Eleven ART-suppressed HIV+ participants (HIV/ART) and 11 HIV-controls matched for age (± 5 years), race, sex, and smoking status were enrolled (Table 1). In each group, 73% of the subjects were male and 91% were African American. Participants had a median age of 53 years and 51 years in the HIV+ and control groups, respectively. To focus on individuals at high risk of SNAEs, HIV+ participants were required to have nadir CD4 counts < 350 cells/µl (median 129 cells/µl; range, 24-233), and at enrollment they had a median CD4 count of 529 cells/µl (range, 342-1062). Participants were on ART for a mean of 7.4 years and virally suppressed with all HIV-1 viral load levels < 200 for 6 months prior to entry and below the lower level of quantitation (< 20 copies/ml) at enrollment. One-fifth of the participants in each group were smokers.

### Monocytes in HIV+ Subjects with Long-Term ART Suppression Have Surface Activation Marker Patterns Similar to HIV- Controls

**Figure 1.**
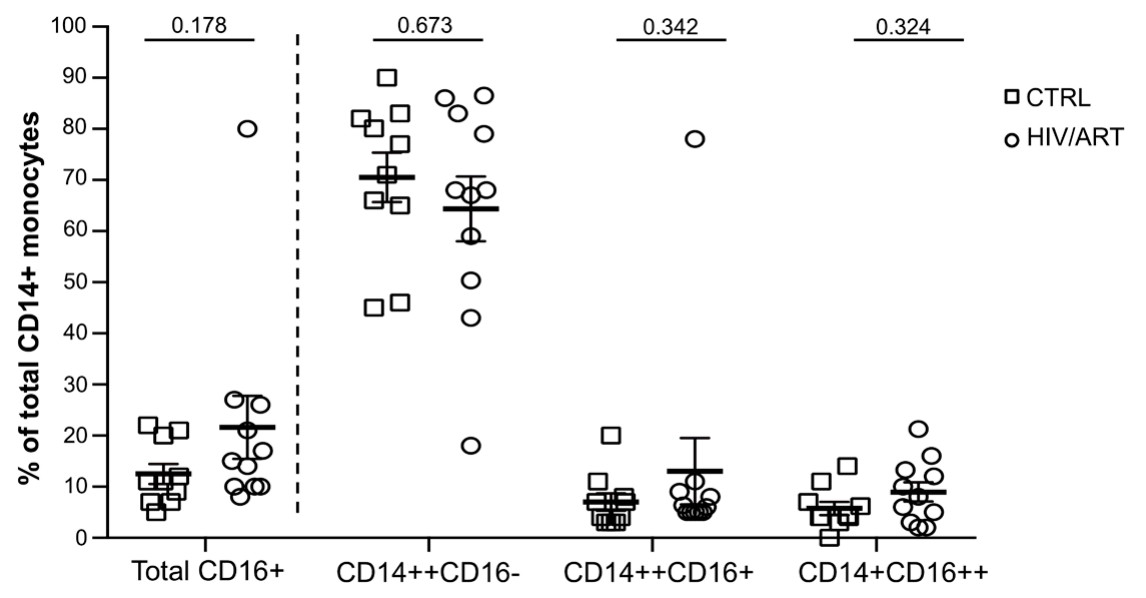
**Proportion of monocyte subsets in HIV/ART individuals is similar to HIV-negative controls.** Freshly isolated PBMCs from HIV+ and HIV-individuals were stained for monocyte surface markers and analyzed by flow cytometry. Monocyte subsets based on CD14 and CD16 surface marker expression in HIV/ART versus matched HIV-negative control individuals is shown. Results are expressed as mean ± SE; *P* value calculated using unpaired Mann Whitney U-test.

There are conflicting reports regarding the impact ART on monocytes [20, 22, 29]. Therefore, we investigated the proportion of monocyte subsets in these long-term-treated but high-SNAE-risk individuals based on CD14 and CD16 surface expression (Figure 1), as CD16+ monocytes have been most frequently associated with inflammatory conditions such as HAND and CVS disease [22, 30]. We compared the classical (CD14++CD16-), intermediate (CD14++CD16+) and non-classical (CD14+CD16++) monocyte subsets [31, 32] in freshly drawn whole blood (Figure 1 and Supplementary Figure 1). The proportion of total CD16+ monocytes was modestly but not significantly higher in HIV/ART than in control subjects (*P* = 0.178). There were no significant differences in the proportion of CD14++CD16+, CD14+CD16++, or CD14++CD16-monocyte subsets.

**Figure 2.**
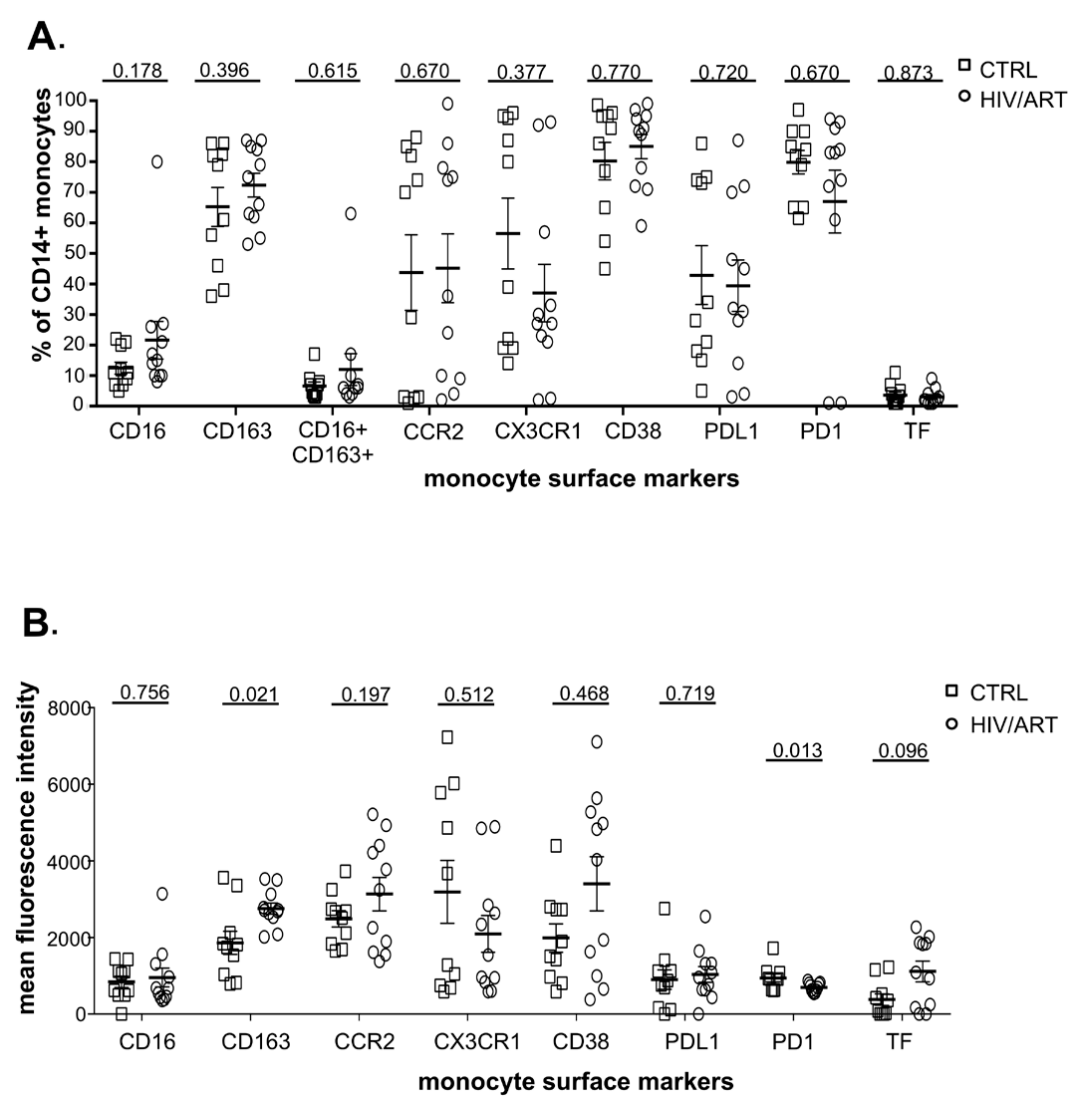
**Proportion and density (MFI) of monocytes expressing surface markers associated with immune activation.** (A) Percentage of CD14+ monocytes expressing monocyte activation-associated markers and (B) Mean fluorescence intensity of marker expression in freshly isolated monocytes from virally suppressed HIV/ART participants and matched HIV-negative controls. Results are expressed as mean ± SE. *P* value is calculated using unpaired Mann Whitney U-test.

We then examined expression of monocyte surface molecules that have been reported to be elevated in HIV infection, particularly CD163, CD16+CD163+ co-expression, and CCR2, the receptor for CCL2/MCP-1 that plays a central role in monocyte recruitment into tissues [33]. We also examined tissue factor (TF), CX3CR1, PDL1, PD1, and CD38, which have also been reported to be dysregulated in monocytes [20, 22, 34–36]. As shown in Figure 2A, HIV/ART participants and healthy controls were not significantly different in the proportions of monocytes expressing these markers. We then investigated expression levels based on mean fluorescence intensity (Figure 2B). Although the proportion of CD163+ monocytes was not different between groups (Figure 2A), CD163 expression based on MFI was significantly greater in HIV/ART participants (*P* = 0.02). TF was also expressed at a modestly higher level on HIV+ compared to control monocytes, although this did not reach significance (*P* = 0.096). By contrast, expression of PD1 was significantly lower on HIV/ART monocytes (*P* = 0.013). The percent TF+ monocytes showed an inverse association with nadir CD4 count that was borderline significant (*P* = 0.057: Spearman correlation), whereas no other monocyte markers were associated with clinical or demographic features (data not shown).

We then asked whether surface marker expression might differ between HIV/ART and control participants specifically within individual monocyte subsets (Supplementary Figures 3 and 4). While there was no difference in the proportion expressing any of the tested markers in monocytes as a whole (Figure 2A), there was a trend toward decreased CX3CR1+ monocytes in HIV/ART classical and intermediate subsets (*P* = 0.074 and 0.08, respectively; Supplementary Figure 3). There were also reductions in the HIV/ART group in CD38+ and PD1+ non-classical monocytes (*P* = 0.058 and *P* = 0.045, respectively), though not in the classical and intermediate subsets. For level of expression based on MFI, which showed significantly greater CD163 and lower PD1 and a trend toward higher TF in the HIV/ART monocytes as a whole (Figure 2B), we saw that these differences were particularly pronounced in specific monocyte subsets (Supplementary Figure 4). CD163 expression was increased only in intermediate and non-classical monocytes (*P* = 0.0008 and 0.006, respectively), a trend toward higher TF expression was seen particularly in the classical monocytes (*P* = 0.07), whereas PD1 showed a trend toward lower expression mainly in the intermediate and non-classical monocytes (*P* = 0.072 for both subsets). In addition, there was significantly lower CD38 expression in HIV/ART monocytes only in the non-classical monocyte subset (*P* = 0.034).

Together, these observations suggest that long-term ART-treated HIV-infected participants have largely normalized monocyte subsets, but display modest abnormalities of selective markers consistent with subtle dysregulation.

### Plasma Cytokines That Distinguish ART-Suppressed HIV+ Individuals From Controls

**Figure 3.**
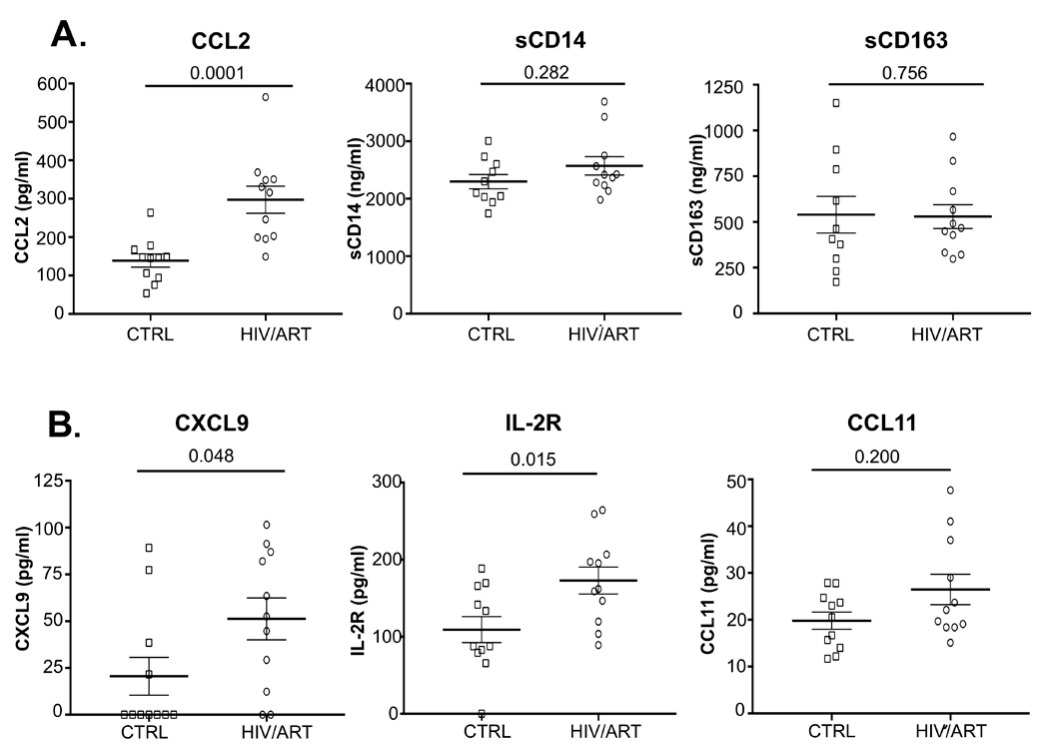
**Elevated levels of several plasma biomarkers in HIV-suppressed individuals compared with matched HIV-negative controls.** (A) Plasma CCL2 (MCP1), sCD14, and sCD163 quantitated by Luminex and ELISA in HIV/ART and uninfected controls (*P* values calculated using unpaired Mann Whitney U-test). (B) Plasma CXCL9 (MIG), IL-2R (sIL-2R), and CCL11 (eotaxin) quantitated by Luminex (*P* values calculated using non-parametric unpaired Mann Whitney U-test).

We then investigated plasma soluble factors in these participants (Figure 3 and Table 2). We were particularly interested in CCL2 (MCP-1), because that is associated with monocyte emigration into the CNS in HAND and vascular tissues in cardiovascular complications [9, 37]. We also examined sCD14 and sCD163 as indicators of monocyte activation that are elevated in untreated infection and persist despite ART suppression in some studies [38–40]. As shown in Figure 3A, HIV/ART subjects had CCL2 levels that were twice that of healthy controls (*P* < 0.0001). In contrast, sCD14 and sCD163 were not different between the groups.

**Table 2.** Plasma cytokines and inflammatory markers in HIV/ART individuals and matched HIV-controls.

Soluble Marker^a^	Control (n = 11)	HIV/ART (n = 11)	*P* value (unpaired Mann-Whitney test)
**CCL2 (MCP1)**	139 (54–264)	298 (149–565)	0.0001*
**CXCL9 (MIG)**	21 (0–89)	51.3 (0–102)	0.048*
**IL-2R**	109 (0–188)	173 (89–264)	0.015*
**CCL11 (Eotaxin)**	20 (12–28)	27 (15–48)	0.200
**CCL3 (MIP-1α)**	29 (0–127)	24 (0–134)	0.843
**CCL4 (MIP-1β)**	98 (0–286)	87.2 (29–399)	0.532
**CXCL10 (IP-10)**	19 (8–46)	34 (1.4–160)	0.358
**CCL5 (RANTES)**	1035 (750–1406)	1016 (569–1588)	0.947
**IL-12**	76 (33–110)	82 (16–122)	0.511
**IL-6**	3.0 (0–8.2)	3.5 (0–12)	0.742
**IFN-α**	17 (0–84.4)	19 (0–85)	0.528
**IFN-g**	26 (8–157)	13 (7–16)	0.645
**IL-1β**	8.0 (0–35)	4.2 (0–46)	0.553
**TNF-α**	3.0 (0–24)	2.0 (0–10)	0.739
**IL-10**	1.4 (0–8)	3.0 (0–6.0)	0.290
**IL-1Ra**	119 (54–230)	126 (0–271)	0.693
**IL-13**	5.0 (0–10)	3.0 (0–11.4)	0.076
**IL-2**	12 (0–46)	3.0 (0–30)	0.203
**IL-5**	6.0 (0–49)	2.0 (0–21)	0.729
**IL-7**	0 (0–4)	0	-
**IL-8**	0	3.0 (0–18)	-
**IL-4**	11 (0–23)	6.0 (0–41)	0.052
**IL-15**	187 (0–733)	64 (0–705)	0.304
**IL-17**	0	0	-
**GM-CSF**	0 (0–3)	1.0 (0–3)	0.664
**sCD163 (ng/ml)**	540 (172–1150)	529 (298–966)	0.756
**LBP (μg/ml)**	24 (10–39)	25 (17–29)	0.751
**sCD14 (ng/ml)**	2297 (1744–3006)	2571 (1982–3689)	0.282

a Soluble markers were measured by Luminex technology except for sCD163, sCD14, and LBP (LPS binding protein), which were measured using ELISA. Values below the level of detection for each analyte are designated as zero. All values are in pg/ml unless stated otherwise. Reported values are mean (range).

* *P* < 0.05 was considered to be significant.

We then examined an extended panel of cytokines, chemokines, and other markers (Figure 3B and Table 2). Soluble IL-2R (sIL2R) and CXCL9 (MIG) were significantly elevated in HIV/ART participants (*P* = 0.015 and *P* = 0.048, respectively). CCL11 (eotaxin-1) was modestly but non-significantly elevated (*P* = 0.200). Of interest, LBP (LPS binding protein) was not elevated in HIV/ART compared to control.

**Figure 4.**
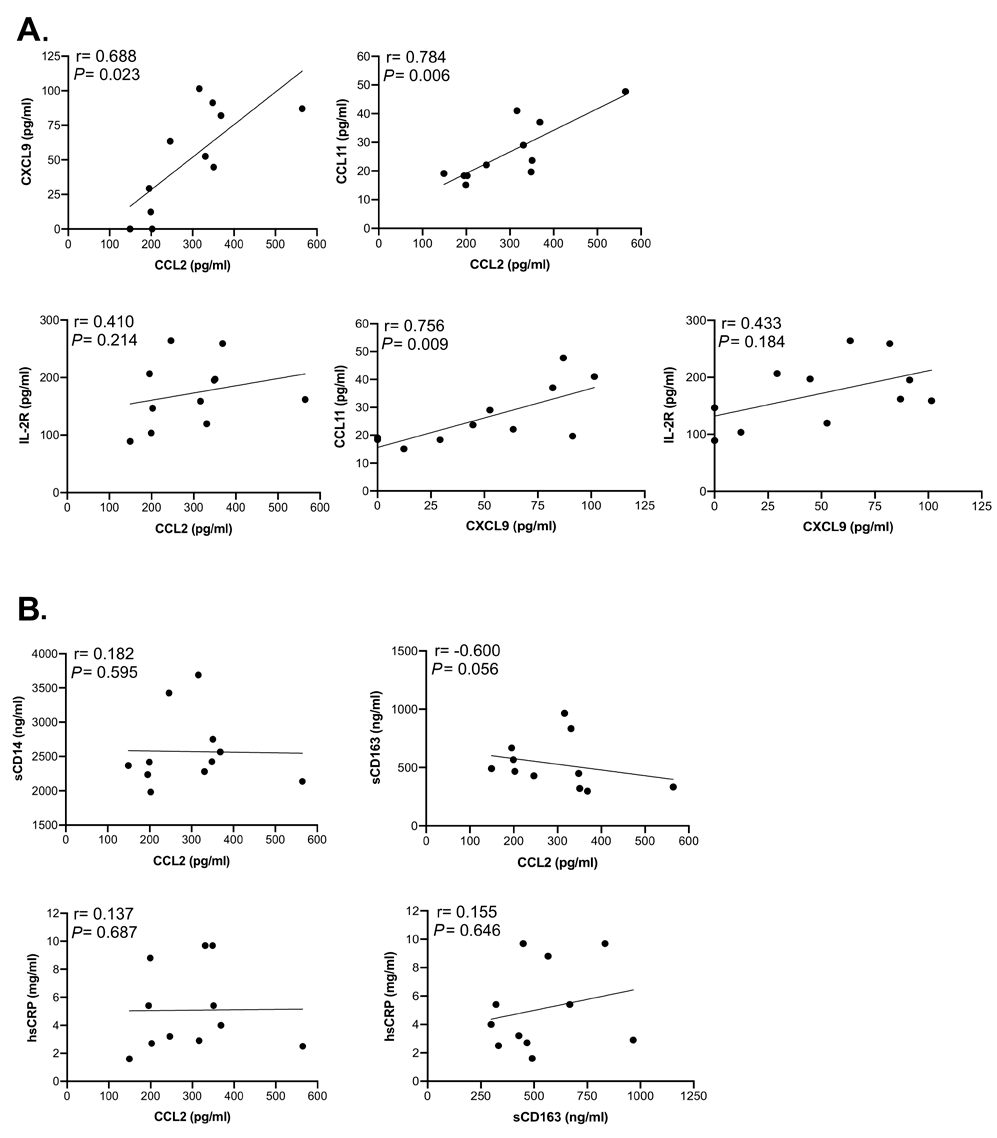
**Correlation analysis between soluble markers in HIV/ART participants shows significant associations.** Spearman correlation test was used to assess the relationship between the measured parameters in HIV/ART subjects. (A) Correlations between CXCL9, CCL2, CCL11, and IL-2R (sIL-2R). (B) Correlations between sCD14, CCL2, sCD163 and hsCRP. The correlation coefficient and significance values for each comparison are shown over each graph.

**Supplementary Figure 2.**
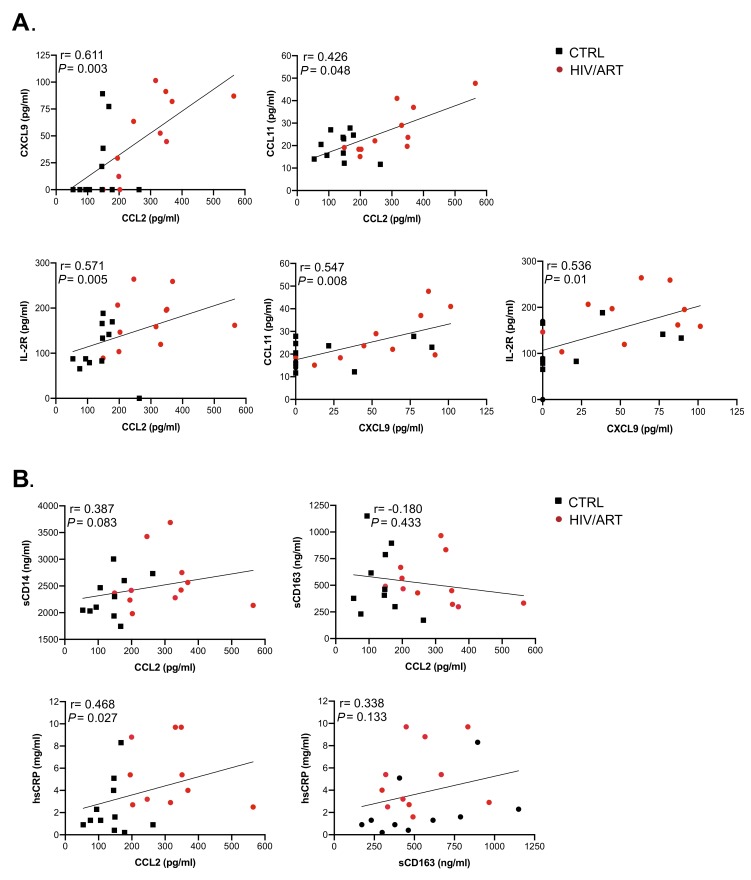
**Correlation analysis between soluble markers combining HIV/ART and HIV-negative participants.** A Spearman correlation test was used to assess the significance of correlations between the measured parameters combining HIV/ART and HIV-negative participants. (A) Correlations between CXCL9, CCL2, CCL11, and IL-2R (sIL-2R). (B) Correlations between sCD14, CCL2, sCD163, and hsCRP. The correlation coefficient and significance values for each comparison are shown over each graph.

**Supplementary Figure 3.**
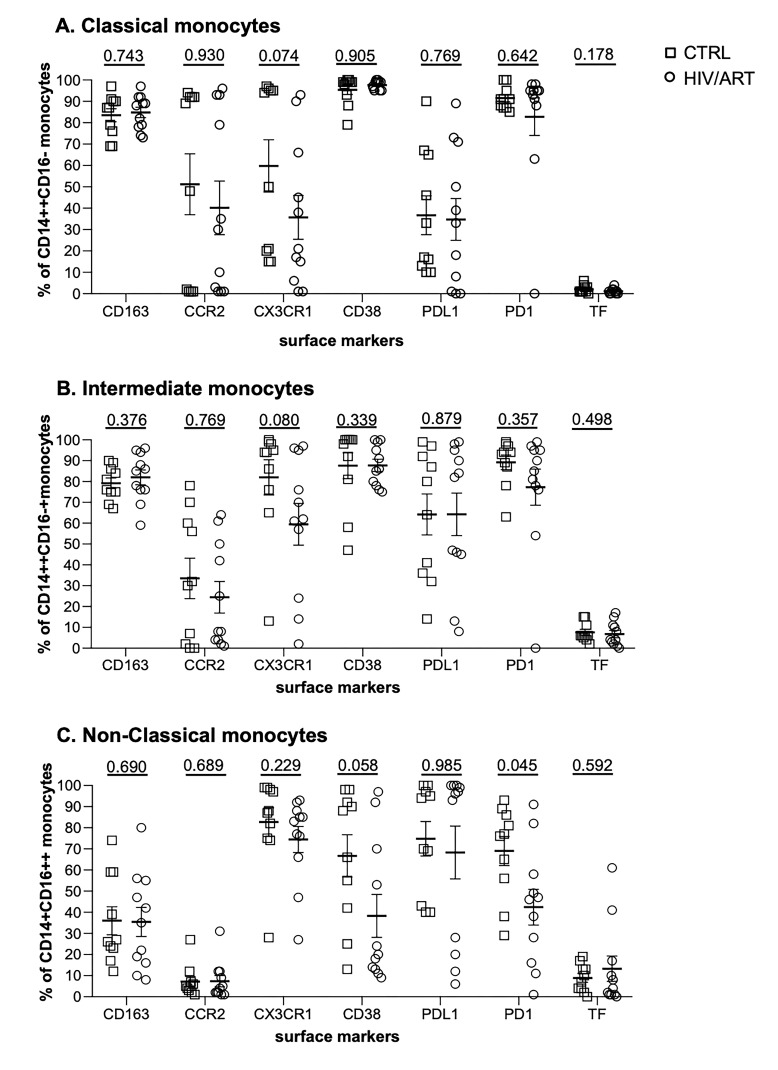
**Proportion of monocyte subsets expressing surface markers.** (A) Percentage of classical (CD14++CD16-), (B) intermediate (CD14++CD16+), and (C) non-classical (CD14+CD16++) monocyte subsets from virally suppressed HIV/ART participants and matched HIV-negative controls expressing the indicated markers. Results are expressed as mean ± SE. *P* value is calculated using unpaired Mann Whitney U-test.

**Supplementary Figure 4.**
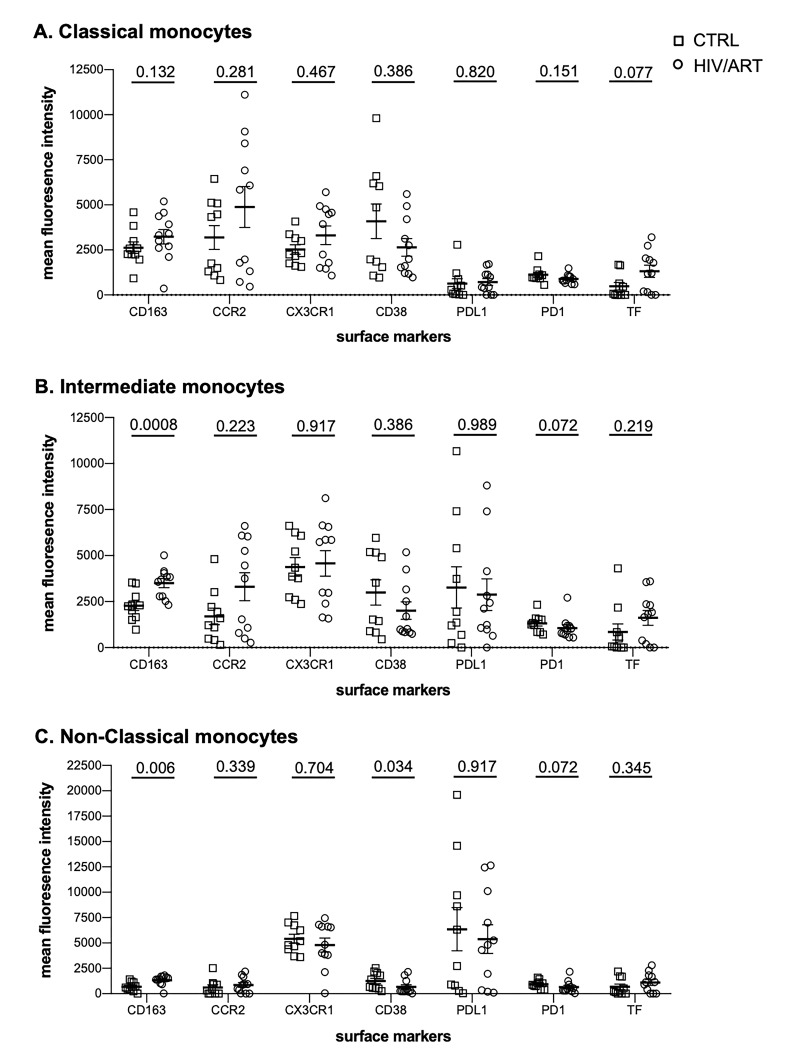
**Mean fluorescence intensity of surface markers on monocyte subsets.** MFI of indicated markers on (A) classical (CD14++CD16-), (B) intermediate (CD14++CD16+), and (C) non-classical (CD14+CD16++) monocyte subsets from virally suppressed HIV/ART participants and matched HIV-negative controls. Results are expressed as mean ± SE. *P* value is calculated using unpaired Mann Whitney U-test.

To identify relationships between soluble factors, we analyzed the correlation between molecules that were elevated in HIV+ participants (Figure 4A). CCL2 was strongly correlated with CXCL9 (*P* = 0.023) and CCL11 (*P* = 0.006) but not with sIL-2R (*P* = 0.214). CXCL9 also correlated with CCL11 (*P* = 0.009) but not sIL-2R (*P* = 0.184). In contrast, sCD14, and hsCRP did not correlate with CCL2, nor did sCD163 with hsCRP, whereas sCD163 and CCL2 showed a modest, although non-significant correlation (*P* = 0.056) (Figure 4B). When correlations between these markers were assessed by considering both HIV/ART and HIV-negative controls (Supplementary Figure 2), the relationships were even more significant between CXCL9 and CCL2 (*P* = 0.003), sIL-2R and CCL2 (*P* = 0.005), and CCL11 and CXCL9 (*P* = 0.008). In addition, relationships between CCL11 and CCL2, sIL-2R and CXCL9, and hsCRP and CCL2 were also significant (*P* = 0.048, *P* = 0.01 and *P* = 0.027, respectively). Together, these observations identify CCL2, CXCL9, CCL11, and sIL2R as reflecting persistent HIV/ART immune dysregulation.

**Figure 5.**
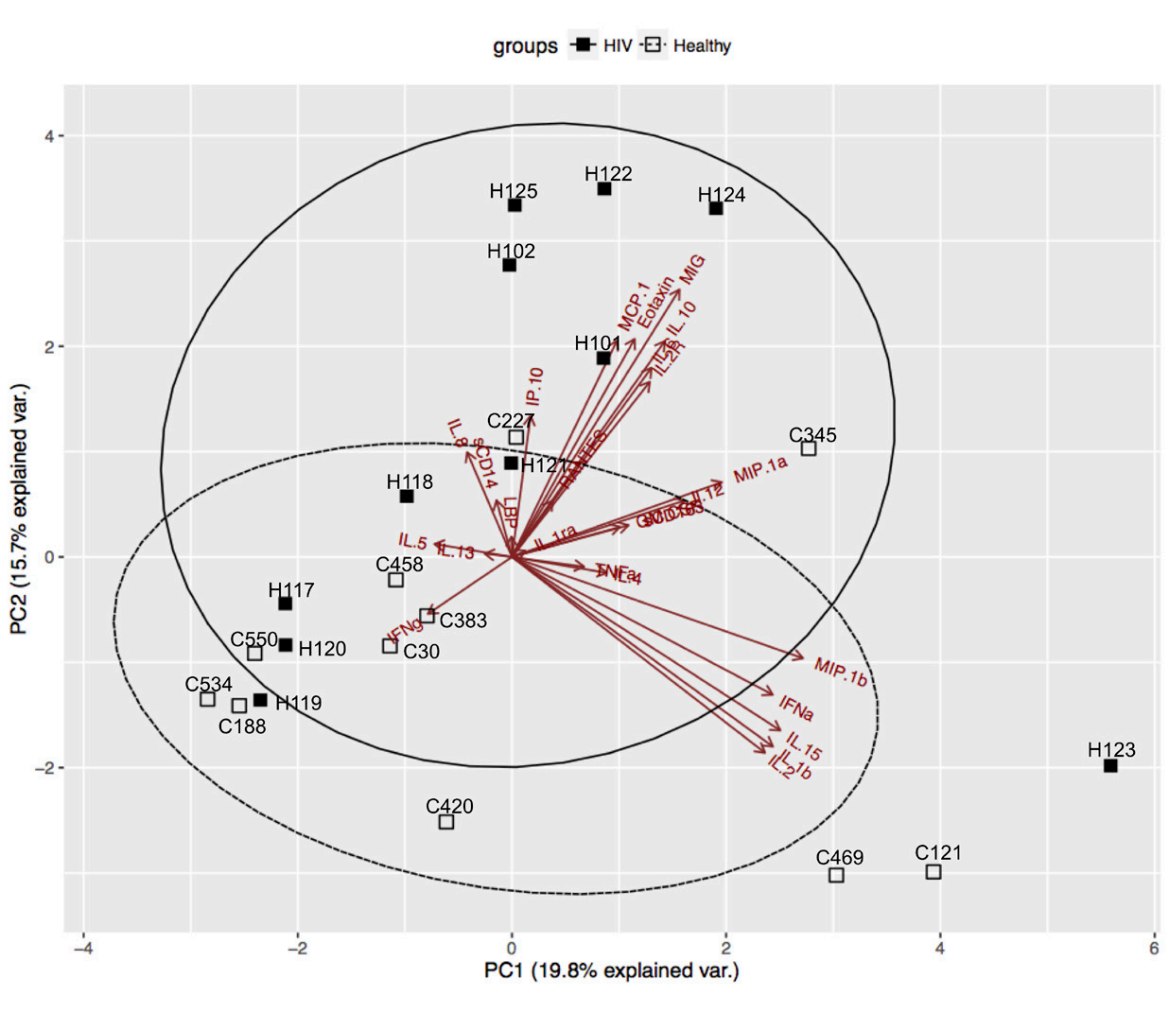
**Principal Component Analysis (PCA) of soluble factors identifies subgroups within HIV/ART group.** PCA of all cytokines and chemokines (Table 2) identified a subgroup of HIV/ART participants (H101, H102, H122, H124, H125) based on increased levels of CCL2 (MCP1), CCL11 (Eotaxin), CXCL9 (MIG), IL-2R, IL-10, IL-6, and CXCL10 (IP-10) and were separated from the other HIV/ART participants (H117, H118, H119, H120, H121), who clustered with HIV-negative controls.

We then applied principal component analysis (PCA) as an unsupervised data reduction tool to visualize the soluble biomarkers and, in addition, identify whether groupings were evident within the HIV/ART participants (Figure 5). As expected, PCA analysis confirmed that the profile of soluble biomarkers differed in HIV/ART participants and HIV-negative controls. The differences were driven largely by CCL2 (MCP-1), CXCL9 (MIG), CCL11 (eotaxin-1), sIL-2R, IL-10, CXCL10 (IP-10), and IL-6. Of these, CCL2, CXCL9, and sIL-2R were identified as significantly increased in HIV/ART versus HIV-negative controls, whereas the remaining biomarkers showed non-significant increases in HIV/ART group (Table 2).

The PCA analysis demonstrated that the distinct HIV/ART profile resulted mainly from a subgroup of 5 HIV/ART participants, while 6 others clustered with controls. These 5 HIV/ART participants (H122, H124, H125, H101, H102) were significantly older than the HIV/ART participants that grouped with the controls (mean ± SE: 57.6 ± 1.6 years versus 36.4 ± 2.4 years respectively; *P* = 0.012, Mann-Whitney test). There were no other differences between the two HIV/ART subgroups with regard to CD4 nadir, current CD4 count, CD4/CD8 ratio, D-dimer, or other factors. Thus, these observations suggest that age is an important factor driving the inflammatory cytokine profile, and that older HIV/ART participants show a pattern that is distinct from both younger HIV/ART participants and HIV-negative controls of the same age group.

### Differentially Expressed Genes in Monocytes of HIV/ART Compared with Control Participants

**Figure 6.**
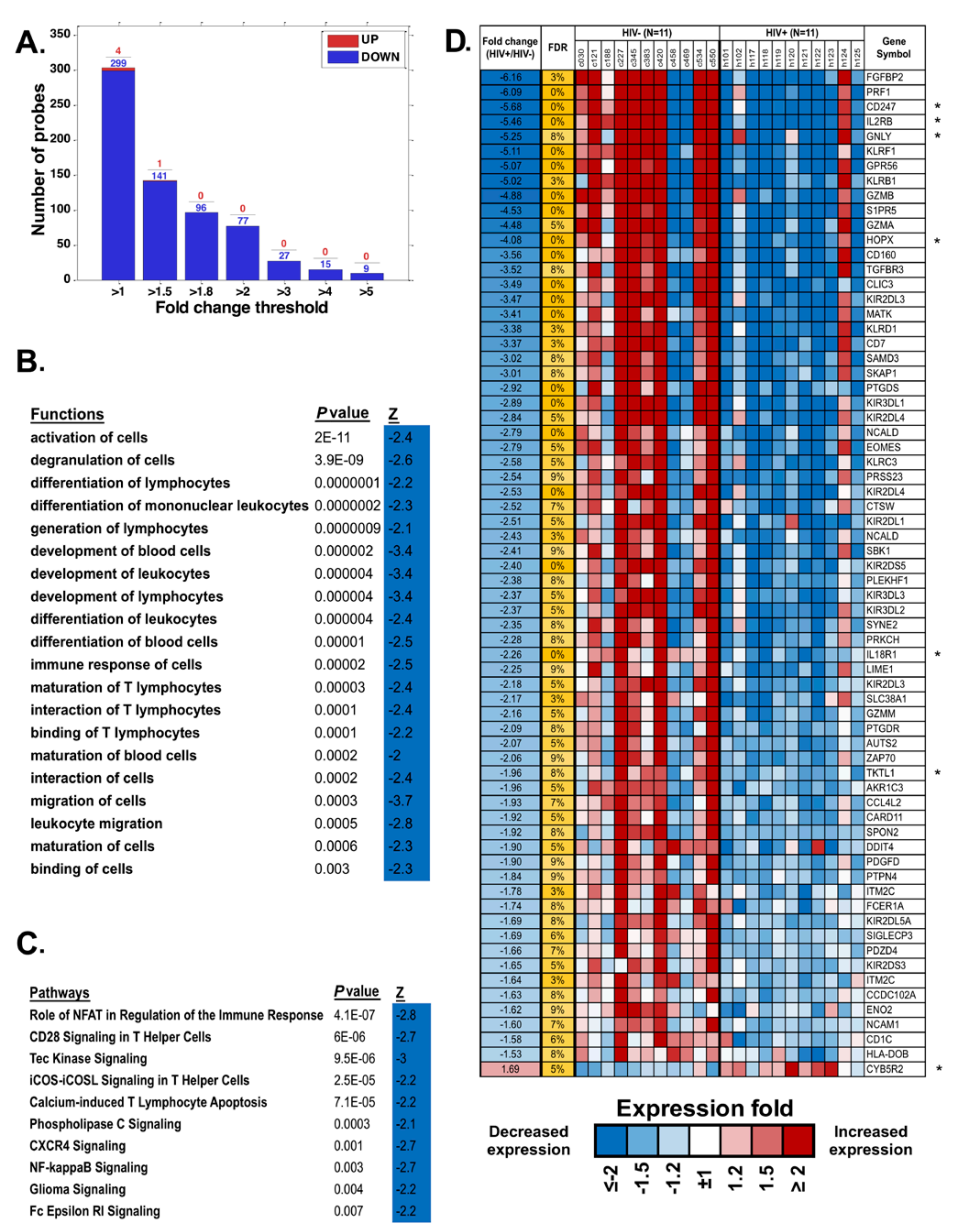
**Monocyte gene expression dysregulation in HIV/ART.** (A) Fold change of differentially expressed monocyte genes (FDR < 20%) in HIV/ART versus HIV-controls. Most of the significant genes are downregulated in HIV/ART group (blue). (B, C) Functions (B) and pathways (C) that are significantly enriched (*P* < 0.01) among genes changed in HIV+/ART individuals compared with those in healthy controls, with predicted activation Z score calculated by IPA indicating inhibition of functions and pathways in HIV/ART subjects. (D) Heat map of top differentially expressed genes in HIV/ART versus HIV-control monocytes (FDR < 10%; fold change > 1.5). Expression levels are shown as fold change in HIV/ART compared with average across samples and sorted by fold change between HIV+/control. Range of colors (blue to red) reflects range of expression for each sample (low to high) and corresponds to log_2_ ratio vs average across all samples. FDR: false discovery rate; Z: z-score; *P*-value: nominal *P* value of the enrichment; Gene symbols with * indicate genes in the HIV interaction database.

We analyzed gene expression patterns of purified monocytes from HIV/ART participants and matched controls using Illumina microarrays. Comparisons between the two groups identified 303 significantly differentially expressed probes (FDR < 20%). Unexpectedly, the great majority of these were downregulated in HIV+/ART participants (Figure 6A). Ingenuity Pathway Analysis (IPA) of the significantly differentially expressed probes identified functions (Figure 6B) and pathways (Figure 6C) significantly affected in HIV/ART participants, all of which were predicted to be inhibited, based on the direction of change of involved genes. Affected functions included cellular activation, cell-to-cell interaction and migration, cell development, and immune response of cells, while pathways included NFAT regulation of immune response and NF-κβ signaling, among others.

We then focused on the top 76 probes, representing 68 differentially expressed genes (DEGs), that were significant at FDR < 10% and changed 1.5-fold (Figure 6D) or more. Of these DEGs, 67 were downregulated and only one was upregulated in HIV/ART participants relative to controls. Overlap of these genes with significantly enriched functions (Figure 6B) was broadly categorized into groups related to inflammatory response, immune cell trafficking, cellular development and cell-to-cell signaling and interaction Table 3). Several of the DEGs (Table 3, in bold) are associated with multiple functional categories.

**Table 3.** Functional classification of differentially expressed monocyte genes in HIV/ART participants compared with HIV-controls.

**Inflammatory response** Immune response of cells Degranulation of cells	**CD247, CARD11, IL18R1, KLRB1, KLRF1, FCER1A, PTGDS, PRF1**, CD7, **NCAM1, IL15, PTGDR**, PRSS23, PTPN4
**Cellular movement, immune cell trafficking** Migration of cells Leukocyte migration	SKAP1, **CD247, EOMES, FCER1A, PRF1, IL2RB, ZAP70, PTGDS, PTGDR, GNLY, SPON2**, SIPR5, **NCAM1, GZMB**, TGFBR3, MATK, PDGFD, SYNE2, **IL15**
**Cellular Development** Development of lymphocytes, leukocytes, blood cells Differentiation of lymphocytes, leukocytes, blood cells Generation of lymphocytes Maturation of lymphocytes, blood cells	**CD247, EOMES, IL15, IL2RB, ZAP70, CARD11**, PRKCH, **GNLY, IL18R1**, RPA1
**Cell-to-Cell Signaling and Interaction** Interaction of T lymphocytes Interaction of cells Activation of cells Binding of cells Binding of T lymphocytes	CD7, **CD247, FCER1A, PRF1, IL2RB, ZAP70, CARD11, KLRB1, KLRD1**, KLRF1, **GNLY**, SIPR5, **NCAM1, GZMB**, GZMA, TGFBR3, DDIT4, **IL18R1**, KIR3DL1, **SPON2**, CD1C, **IL15**, SKAP1

Gene Symbols enriched in HIV/ART compared with controls are shown; genes in bold are enriched in more than one category. All genes listed are downregulated in HIV/ART individuals.

We then used the Database for Annotation, Visualization and Integrated Discovery (DAVID) to identify DEGs overlapping with genes in the HIV Interaction Database [41, 42]. Seven of these 68 DEGs (10%; identified with * in Figure 6D) are also identified in the HIV interaction database (PRF1, CD247, IL2RB, GZMA, PRKCH, ZAP70, and HLA-DOB) as having protein-protein interactions. Thus, HIV/ART monocyte dysregulation also included genes that are involved in host-virus interactions. Because the proportion of monocytes actually infected *in vivo* is very small [43], this observation likely suggests indirect rather than direct effects of HIV infection on these genes.

**Figure 7.**
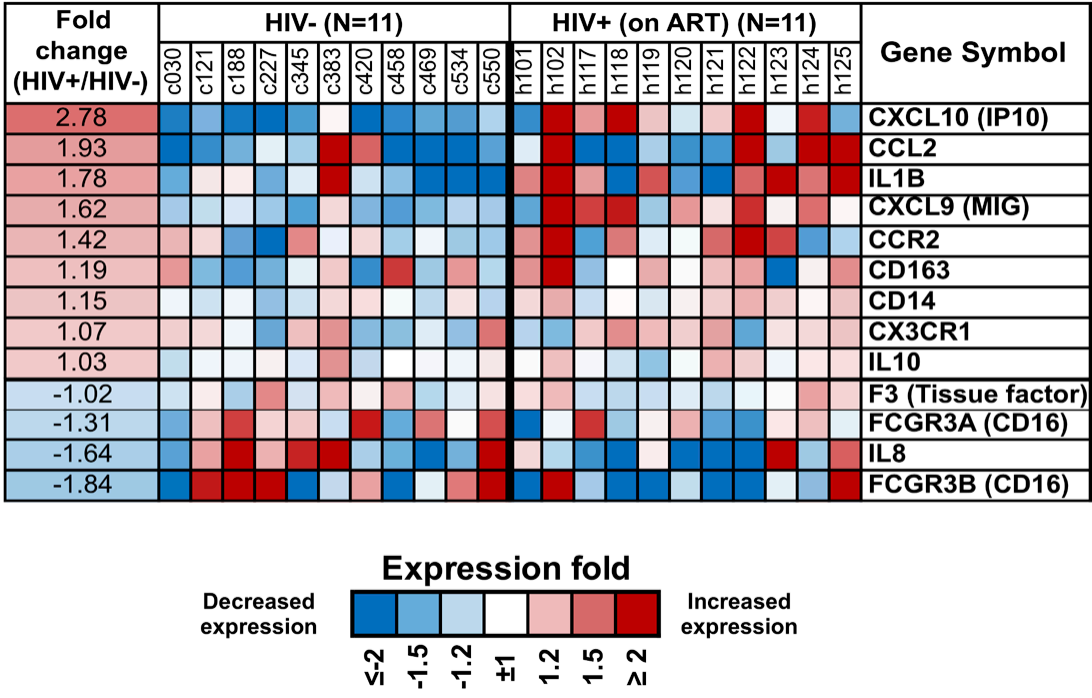
**Differential expression of monocyte genes associated with immune activation in people with HIV based on a priori knowledge.** Heat map display of the microarray gene expression data showing genes coding for monocyte surface receptors and soluble factors in HIV/ART participants versus matched HIV-negative controls. Expression levels are shown as fold change in HIV/ART compared to control arranged in descending order of expression. Range of colors (blue to red) shows range of expression (low to high). Genes showing > 1.5-fold change had nominal *P* value < 0.01.

Next, we specifically queried expression in purified monocytes of an *a priori* set of genes for surface or secreted molecules that have been associated with persistent immune activation in HIV/ART subjects (Figure 7) [9, 13], recognizing that soluble factors might be produced by multiple other cell types as well as monocytes. Results showed heterogeneity between participants, both in the HIV/ART and control groups. However, HIV/ART monocytes generally showed elevated expression of CXCL10, CCL2, CCR2, and CXCL9, and decreased CD16 and IL-8, compared with control participants. While these genes did not reach the FDR < 10% threshold criteria for broader gene analysis, CXCL10, CCL2, IL-1B, CXCL9, IL-8, and CD16 showed > 1.5-fold differences and had nominal *P*-values < 0.01.

### GSEA to Characterize Monocyte Dysfunction in HIV+/ART Group.

While enrichment by IPA provided a set of functions and pathways affected in HIV+/ART participants, it was focused only on statistically significant genes and ignored more subtle differences between the groups. To obtain a more comprehensive picture of the affected pathways and functions, we used Gene Set Enrichment Analysis (GSEA), which employs the whole gene expression dataset ranked in order of difference between the groups, rather than individual genes identified using a pre-specified threshold. Of the categories from KEGG, BioCarta, PID, and REACTOME databases, there were 107 gene sets that were significantly enriched at a nominal *P* < 0.05 and 43 gene sets at *P* < 0.01. A total of 16 gene set pathways were significant at FDR < 25%, most of which were immune-related pathways (Table 4).

**Table 4.** Enriched pathways in monocytes of HIV/ART individuals by GSEA.

Gene set name	GS size	NOM *P*-value	FDR (%)	Core enriched genes contributing to pathway enrichment
KEGG GRAFT VERSUS HOST DISEASE	38	0.000	4.0	PRF1, GZMB, KLRD1, KIR2DL3, KIR2DL1, KIR3DL2, KIR3DL1, HLA-DOB, KIR2DL5A, FASLG, KIR2DL2, IFNy, IL1B, HLA-DOA, HLA-DMB, KLRC1, CD28
PID CD8 TCR DOWNSTREAM PATHWAY	50	0.000	2.5	IL2RB, PRF1, CD247, GZMB, EOMES, STAT4, FASLG, PTPN7, CD3E, PRKCQ, IFN7, CD8A, FOS, CD3G, JUN, EGR1, BRAF, IL2RG, CD8B, IFNAR1
BIOCARTA DC PATHWAY	16	0.000	2.4	CD7, CD2, TLR7, TLR4, CD5, IFN7, TLR2, CD40, TLR9
BIOCARTA IL12 PATHWAY	20	0.001	2.9	IL18R1, CD247, STAT4, CXCR3, CD3E, IFN7, CD3G, JUN, JAK2
PID IL12 STAT4 PATHWAY	29	0.000	3.4	IL18R1, PRF1, CD247, STAT4, CD3E, IFN7, FOS, CD3G, JUN, CD28, IL18RAP, STAT3, CD3D, TBX21
PID IL12 2PATHWAY	58	0.000	3.8	IL18R1, IL2RB, CD247, GZMB, CCL3, EOMES, GZMA, STAT4, FASLG, CD3E, IFN7, STAT1, CD8A, IL1B, FOS, CD3G, null, JAK2, IL2RG, IL18RAP, CD8B, STAT3, NFKB2, ATF2, CD3D, TBX21
BIOCARTA NO2IL12 PATHWAY	16	0.000	3.7	CD247, STAT4, CXCR3, CD2, CD3E, IFN7, CD3G, JAK2
BIOCARTA TOB1 PATHWAY	16	0.000	5.1	CD247, TGFBR3, CD3E, IFN7, CD3G, TGFB3, CD28, TOB1, SMAD4, CD3D
BIOCARTA STATHMIN PATHWAY	16	0.003	9.1	CD247, CD2, PRKAR2A, CD3E, CD3G, MAPK13, CAMK4, CAMK2B, CD3D
REACTOME IMMUNOREGU-LATORY INTERACTIONS BETWEEN A LYMPHOID AND A NON LYMPHOID CELL	63	0.000	14.5	CD160, CD247, KLRD1, KIR2DL3, KIR2DL4, KIR2DL1, KIR3DL2, KIR3DL1, CD81, CD96, KIR2DL2, CD3E, CD8A, CD200R1, CD3G, CD40, KLRC1, KIR2DS1, KLRK1, CD8B, ITGAL
REACTOME HS GAG BIOSYNTHESIS	20	0.004	14.2	EXT1, GPC4, NDST2, HS3ST1, GLCE, HS6ST1, NDST1, SDC2, AGRN, EXT2
REACTOME DEFENSINS	21	0.004	15.0	TLR2, DEFA3, DEFA4, DEFA1, DEFB103B, CCR6, TLR1, CCR2, DEFA1B
KEGG ANTIGEN PROCESSING AND PRESENTATION	74	0.000	15.2	KIR2DS5, KLRD1, KIR2DL3, KIR2DL4, KLRC3, KIR2DL1, KIR3DL2, KIR3DL1, HLA-DOB, KIR2DL5A, KIR2DL2, CD8A, HLA-DOA, HLA-DMB, CTSS, TAP1, KLRC1, KIR2DS1, CD8B, CIITA, LTA, KLRC4, HLA-DQA1
KEGG ETHER LIPID METABOLISM	26	0.003	16.3	PAFAH1B2, PAFAH1B3, PPAP2B, PLA2G4A, LPCAT4, PLA2G7, AGPS, PLA2G6, LPCAT1, PLA2G4B, PLD1, PAFAH1B1, PLA2G12B, PA-FAH2, PLA2G2F
KEGG O GLYCAN BIOSYNTHESIS	21	0.004	18.5	GCNT1, GALNT11, C1GALT1, ST3GAL2, C1GALT1C1, GALNT1, B4GALT5, GALNT3, GALNTL1, GALNT7, GALNT10, B3GNT6, GCNT3
REACTOME HOMOLOGOUS RECOMBINATION REPAIR OF INDEPENDENT DOUBLE STRANDED BREAKS	15	0.016	19.5	RAD50, LIG1, MDC1, BRIP1, NBN, RPA3, RPA1

GS size: Gene set size (number of genes); NOM p-value: nominal *P*-value; FDR: false discovery rate. Shown are processes enriched at FDR < 25%.

**Figure 8.**
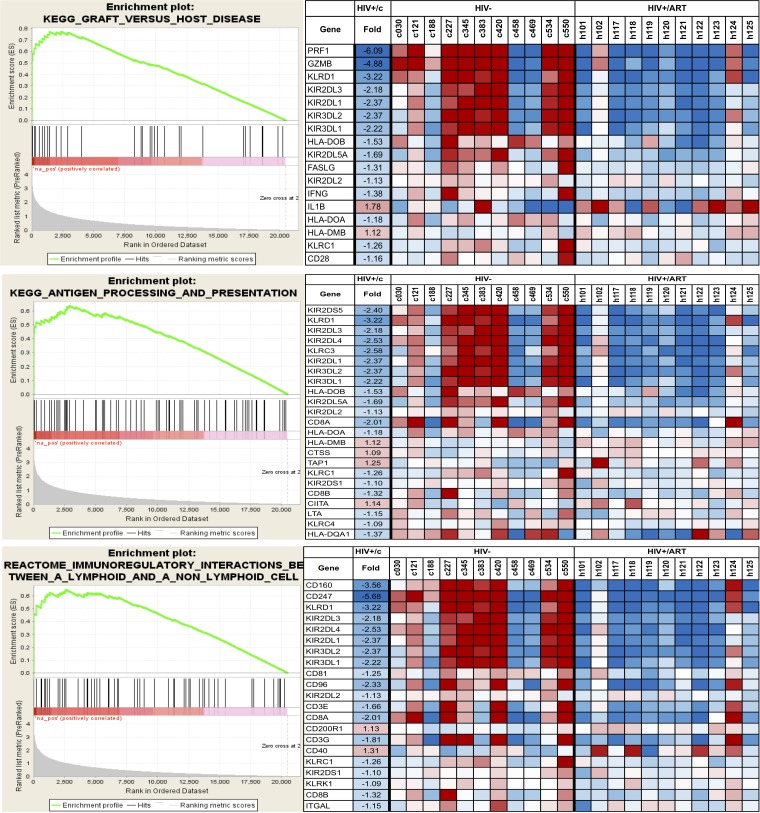
**Selected pathways affected in the monocytes of HIV/ART participants versus HIV-negative controls.** Data shown are for three KEGG immune-related pathways. Left panels: Enrichment plots for monocytes from HIV/ART group. The bottom portion of the plot shows the value of the ranking metric (y-axis) moving down the list of ranked genes (x-axis). The ranking metric measures a gene's correlation with a phenotype. Genes most correlated with HIV/ART have the highest positive ranking metric score. Middle portion of the plot shows location of the genes from the respective pathways within the ranked list. Top of the plot shows enrichment score (ES) for the gene set as the analysis walks down the ranked list. The score at the peak of the plot is the ES for the gene set and genes appearing before or at the peak are defined as core enrichment genes for each set. Right panels: Heat map of core enrichment genes corresponding to respective enrichment plots. Rows: genes; columns: samples; range of colors (red to blue): range of expression (high to low).

Since monocytes are key antigen-presenting cells, one of the enriched pathways of interest is the Antigen Processing and Presentation pathway (Table 4). This pathway was earlier reported to be upregulated in monocytes of HIV+ viremic individuals compared with HIV/ART-suppressed individuals [44]. Our data suggest that in participants who have chronic ART-suppressed HIV/, several genes in this pathway continue to be dysregulated. Strikingly, however, the core-enriched genes in this pathway are mostly downregulated compared with control participants (Figure 8). A similar pattern was seen in two other pathways identified (graft-versus-host disease [GVHD]; Immunoregulatory Interactions Between a Lymphoid and a Non-Lymphoid cell) (Table 4; Figure 8), which have in common with the Antigen Presentation Pathway several of the enriched genes. Finally, as shown in Table 5, several biological processes and functions were observed to be down-regulated in the HIV/ART group. Notable among them are the genes associated with the gene ontology terms cellular defense response, cytokine secretion, and JAK-STAT cascade.

**Table 5.** Overrepresentation of biological processes and molecular functions by GSEA in monocytes from HIV/ART participants.

Gene set name	GS size	NOM *P*-value	FDR (%)	Contributing core enrichment genes
CELLULAR DEFENSE RESPONSE	50	0.000	18.3	PRF1, CD160, CXCL9, KIR2DL4, KLRC3, KIR3DL2, FAIM3, GNLY, ITK, LGALS3BP, CCR6, UMOD, CCR2, MNDA, KLRG1, CCR9, KLRC4, CCR3, LY96, CLEC5A, ADORA2A
AXON GUIDANCE	15	0.006	18.7	SPON2, SLIT2, FEZ2, SLIT1, NRP1, KAL1, SEMA4F
SH2 DOMAIN BINDING	15	0.005	19.5	IRS1, NUP62, SIT1, LAX1, JAK2, ARHGAP5
DI TRI VALENT INORGANIC CATION TRANSMEMBRANE TRANSPORTER ACTIVITY	19	0.003	19.5	IPTR3, ATP2B4, ATP2A2, SLC31A2, ITPR1
CARBON OXYGEN LYASE ACTIVITY	23	0.003	20.9	ENO2, EHHADH, HADHB, CA5B, CA11, NTHL1, ENO3
POSITIVE REGULATION OF TRANSCRIPTION FACTOR ACTIVITY	19	0.006	21.1	CARD11, NLRC3, PRKCQ, NOD2, CEBPG, PRDX3, NFAM1
SERINE HYDROLASE ACTIVITY	29	0.001	21.2	GZMB, GZMA, PROC, DPP4, PREP, CFD, CTSG, PRSS36, F12, APEH, ACHE, CPD
HYDRO LYASE ACTIVITY	19	0.004	22.5	ENO2, EHHADH, HADHB, CA5B, CA11, ENO3
POSITIVE REGULATION OF TRANSLATION	29	0.000	22.7	IRF4, BOLL, TLR7, SAMD4A, TLR4, TLR6, 1L29, LTB, TLR9, IL27, TLR1, CD28, ELANE, EB13, CEBPG, AZU1
SERINE TYPE ENDOPEPTIDASE ACTIVITY	24	0.001	23.7	GZMB, GZMA, PROC, PREP, CFD, CTSG, PRSS36, F12, APEH
CYTOKINE SECRETION	16	0.016	23.9	CADM1, ABCA1, NLRC4, CARD8, NOD2, SRGN
SERINE TYPE PEPTIDASE ACTIVITY	28	0.003	24.2	GZMB, GZMA, PROC, DPP4, PREP, CFD, CTSG, PRSS36, F12, APEH, CPD
JAK STAT CASCADE	28	0.009	25.3	NMI, STAT4, F2R, CLCF1, STAT1, IL29, STAT2, CCR2, IFNAR1, STAT5B, STAT3, CCL2, SOCS2, HCLS1, IL20, IL12A, NF2, FGFR3, PIAS1, SOCS6, SOCS3, PIGU

GS size: Gene set size (number of genes); NOM *P*-value: nominal *P*-value; FDR: false discovery rate. Shown are processes enriched at FDR < 25%.

### Monocyte Gene Expression Pattern in HIV/ART Virally Suppressed Subjects Differs from That In Viremic Asymptomatic HIV+ Subjects

We asked whether the aberrant gene expression patterns identified here in HIV/ART participants' monocytes were similar to aberrant monocyte gene expression patterns previously identified in HIV+ untreated individuals who were viremic, but asymptomatic. To do this, we compared the monocyte gene expression in chronic HIV/ART versus control monocytes from our study (which used the Illumina platform containing 29,208 probes representing 20,464 genes) with a previous study comparing monocytic genes from asymptomatic viremic HIV+ participants versus controls (which used custom cDNA arrays with 19,200 probes representing 14,000 genes) (Supplementary Table 1) [45, 46]. An overlapping group of 7,538 probes was present in both array platforms.

**Figure 9.**
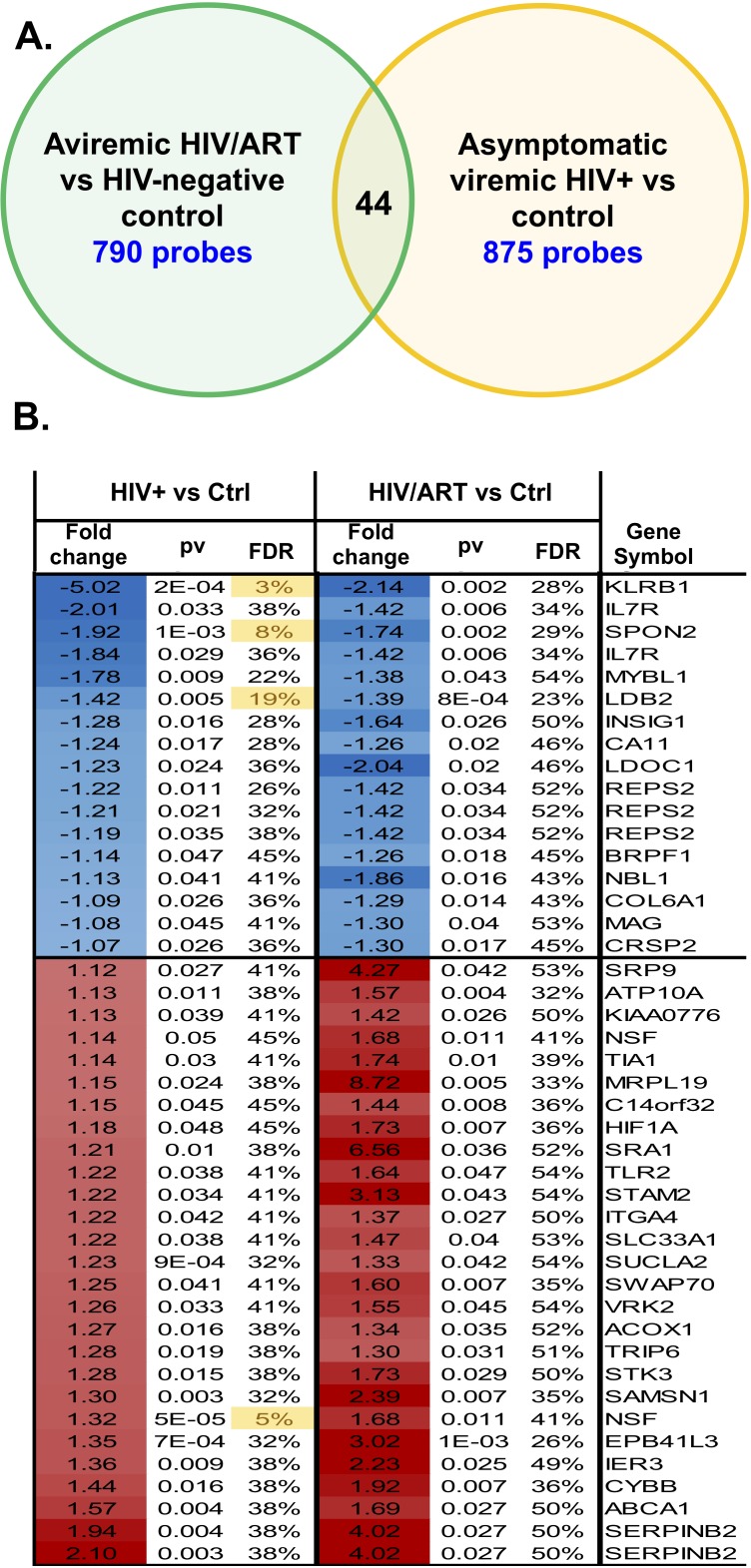
**Monocyte gene expression pattern in aviremic HIV/ART participants is distinct from viremic asymptomatic HIV+ people.** The differences in monocyte gene expression in HIV/ART versus healthy controls determined in the present study (using the Illumina platform; 29,208 probes representing 20,464 genes) was compared to differences in monocyte genes in chronic viremic HIV patients versus healthy controls (using nylon arrays; 19,200 probes representing 14,000 genes) previously reported [45, 46]. There were 7538 probes present in both array platforms. Using a nominal *P* < 0.05 cutoff, 790 probes were different in HIV/ART versus controls, and 875 probes were different in viremic HIV+ versus controls. Of these, 44 probes (39 genes) reached the significance threshold in both comparisons and showed similar directionality, while 56 probes (45 genes) reached the significance threshold in both comparisons but showed opposite directionality. The figure depicts the overlap of probes and genes with similar directionality in the two analyses (HIV/ART compared with controls, and HIV viremic compared with controls).

Applying a nominal *P* < 0.05 cutoff to this group of probes, there were 790 probes that were differentially expressed in HIV/ART versus healthy control participants' monocytes in this study, and 875 probes that were differentially expressed in viremic HIV+ versus control participant monocytes in the prior study (Figure 9A). Among these, only 44 probes showed a similar direction of dysregulated expression in both HIV/ART and HIV+ viremic subjects, representing 39 unique genes (Figure 9B) [45, 46]. Conversely, 56 probes (45 genes) were dysregulated in both HIV+ subject groups compared with healthy controls, but in opposite directions (data not shown). The total number of probes dysregulated in both gene expression analyses (100 probes; 84 genes) was approximately equal to the overlap expected by chance alone (92 probes). Thus, genes dysregulated in the viremic HIV participants as compared with controls are not over-represented in our HIV/ART-versus-control subjects here, which suggests that the pattern of aberrant monocyte gene expression in aviremic HIV/ART participants in our study is fundamentally different from the disruption of monocyte gene expression in viremic HIV+ subjects. Therefore, the monocyte gene expressed abnormalities seen in these long-term ART-suppressed participants reflect a unique pattern, and not simply a less severe version of what is seen in untreated HIV+ subjects' monocytes.

### Validation of Microarray Gene Expression Patterns by qPCR.

The 68 most significant DEGs identified here (Figure 6C) included multiple genes well known to be expressed in monocytes, such as NCAM1 (CD56) [47, 48]. Also identified, however, were several Killer Immunoglobulin-like Receptor (KIR) genes. This finding was unexpected because KIRs are typically associated with NK cells, although KIR gene expression has previously been reported in monocytes [44]. We therefore wished to confirm KIR gene expression in monocytes using qPCR. We isolated mRNA from monocytes from a subset of study subjects (6 healthy controls and 4 HIV/ART), and used RT-qPCR to quantify RNA levels. We selected 2 KIR genes that were significantly downregulated in the HIV/ART group by microarray (KIR3DL1 and KIR3DL2), and 2 other genes (CD247 and IL2RB) that also were observed to be significantly downregulated. As shown in Table 6, qPCR confirmed expression in purified monocyte populations. Furthermore, all genes were downregulated several fold in the HIV/ART subjects compared with controls, consistent with the microarray data. Thus, specific targeted qPCR showed results confirming microarray data for these genes.

**Table 6.** Real time PCR validation of selected differentially expressed genes in HIV/ART versus healthy control participants.

Gene Symbol	Pathways involved	Relative expression HIV/ART (Mean ± SD)	Relative expression HIV-CTRL (Mean ± SD)	Fold difference (HIV/ART vs CTRL)
**KIR3DL1**	Graft versus host disease pathway Immunoregulatory interactions between a lymphoid and a non-lymphoid cell pathway Antigen processing and presentation pathway	0.001 ± 0.002	0.058 ± 0.05	−19
**KIR3DL2**	Graft versus host disease pathway Immunoregulatory interactions between a lymphoid and a non-lymphoid cell pathway Antigen processing and presentation pathway	6.0 ± 4.5	215 ± 237	−36
**CD247**	IL-12 pathway CD8 TCR downstream pathway IL-12 STAT4 pathway Immunoregulatory interactions between a lymphoid and a non-lymphoid cell pathway Stathmin pathway TOB1 pathway NO2IL-12 pathway	0.08 ± 0.10	0.15 ± 0.17	−2
**IL2RB**	IL-12 pathway CD8 TCR downstream pathway	18 ± 19.3	564 ± 931.2	−31

Fold difference was calculated from the mean expression levels for each gene relative to a housekeeping gene (IPO8), and is expressed as a negative value indicating down-regulation of the gene in HIV/ART+ compared to control participants.

## DISCUSSION

We provide evidence here of monocyte and immune dysregulation after long-term ART despite recovery of monocyte subset frequencies and levels of sCD14 compared with HIV-negative control levels. Myeloid cells are proposed to play a central role in the serious non-AIDS events (SNAEs) that are associated with long-term HIV infection despite effective viral suppression. To understand monocyte recovery after ART, we employed complementary approaches of monocyte surface phenotyping, soluble cytokine analysis, and monocyte transcriptional analysis. We focused on subjects with HIV who are long-term ART-suppressed and without any of these comorbid conditions, but who are considered at risk based on initiating ART at low CD4 nadir, older age, and elevated plasma hsCRP. We found that monocyte inflammatory subsets in these individuals were not significantly different from those observed in healthy individuals, and only modest differences were seen in activation surface markers; a number of plasma biomarkers were elevated, which were highly interrelated, and cluster analysis identified a subgroup of subjects with coordinated cytokine elevations; and that multiple monocyte genes and gene pathways were significantly different in HIV/ART compared with control monocytes.

The first notable finding is that the proportions of cells in major monocytes subsets linked to persistent inflammation were not different in our HIV/ART subjects compared with HIV-negative controls. Total CD16+ monocytes as well as intermediate (CD14++CD16+) and non-classical (CD14+CD16++) monocytes were only marginally and not significantly higher in the HIV/ART group. This result is consistent with studies reporting reversal of the monocyte subsets indicative of activation in ART-suppressed individuals [20], but differs from others describing persistent alterations in CD16+ expression despite ART [18, 22]. It is likely that the duration of ART suppression or other aspects of subject selection are responsible for differences among studies. In contrast, we did see subtle abnormalities of surface marker expression, such as increased CD163 and decreased PD1, as well as a trend toward increased tissue factor (TF). TF is of particular interest, as it is thought to be a key driver of SNAEs through activation of coagulation pathway mediators [49]. The aberrant TF expression in these subjects was less marked than has been described in individuals with shorter durations of ART, given the median > 7 years of ART therapy in our subjects. Together, these suggest that following very long-term ART, there is substantial but incomplete normalization of monocyte activation.

Analysis of monocyte subsets revealed that, beyond altered markers seen in HIV/ART monocytes as a whole, several additional markers were altered only in specific subsets. This included decreased CD38 MFI in non-classical monocytes and a trend toward fewer CX3CR1+ classical and intermediate monocytes. CX3CR1 is the receptor for fractalkine, which plays a role in trans-endothelial monocyte migration, and has been reported to be elevated in HIV/ART plasma [22]. Decreased CX3CR1 expression on HIV/ART monocytes has been reported previously (although based on MFI, rather than percentage) [22]. Although we did not measure plasma fractalkine, together, these findings suggest that abnormalities of the CX3CR1/fractalkine axis might contribute to monocyte/macrophage-related comorbidities in virally-suppressed individuals.

The second principal finding is that, alongside the largely though incompletely normalized monocyte profiles, a number of soluble factors associated with persistent immune activation remained elevated. Most striking was CCL2 (MCP-1), which was twice the level of matched healthy controls. CCL2 plays a key role in monocyte recruitment into tissues, including the CNS and vascular wall [50, 51], and is believed to be central in the development of neurological and cardiovascular complications in HIV/ART subjects [9, 52, 53]. Thus, this mechanistically important chemokine is the most prominent abnormal mediator late after viral suppression. In addition, CXCL9 (MIG) and IL2R were also significantly elevated in the HIV/ART subjects as a group. Multiple other soluble factors showed a trend but did not reach statistical significance, which we ascribe to heterogeneity within the HIV subgroup (discussed further, below). In contrast, levels of certain other markers that have been linked to SNAEs, such as sCD14 and sCD163 [16], were increased only marginally or not at all.

The marked elevated CCL2 (MCP-1) levels in these people living with HIV despite long-term viral suppression are consistent with others' evidence implicating the CCL2/CCR2 axis as a central component of persistent immune activation, and a mechanistic role in HIV-associated neurologic, cardiovascular, and metabolic disorders [54–57]. Therefore, inhibiting the CCL2/CCR2 axis might be an attractive approach for prevention or treatment of HIV-associated comorbidities [52, 58]. Small-molecule CCL2 blockers have been developed and studied *in vivo* in other conditions [59, 60]. In HIV infection, studies have investigated CCL2 or dual CCR2/CCR5 blockade *in vitro* and *in viv*o, although with a focus on antiviral effects, rather than inflammation or comorbidities [61, 62]. Thus, the long-term persistence of CCL2 elevations combined with its role in recruiting monocytes into tissues, including brain and vascular tissues, suggest that targeting CCL2/CCR2 should be investigated for prevention or treatment of these comorbidities.

Principal component analysis of these soluble factors revealed that approximately half of the HIV/ART subjects clustered with healthy controls, and clearly delineated a subset of ART-suppressed persons with persistent activation. The HIV outlier cluster was driven not only by the molecules that were significantly elevated in HIV/ART versus healthy subjects (CCL2, CXCL9, and IL2R), but also by CXCL10 (IP-10), CCL-11 (Eotaxin), IL-6, and IL-10, which showed only a trend toward elevation compared with controls. Thus, the control-like cluster and outlier cluster revealed by this analysis explain the heterogeneity that limited statistical power in this relatively small group of HIV/ART subjects. Several of these molecules have been associated with SNAEs and mortality in HIV/ART treated subjects [9, 57, 63]. Subjects in the outlier group did not differ from those that grouped with controls in CD4 nadir, current CD4 count, or other measured factors, but they were significantly older. Consistent with this, the incidence of HIV-associated SNAEs increases with age. Thus, we find a pattern of coordinated plasma cytokine dysregulation that is observed in only a subpopulation of HIV/ART subjects, potentially increasing their risk of SNAEs.

Finally, we used several complementary analytic approaches to interrogate monocyte gene expression in these long-term ART-treated subjects, and identified global dysregulation of multiple inter-related pathways associated with antigen presentation and immune function. The direction of dysregulation among these pathways was almost uniformly downregulation. This finding is consistent with the long-standing notion that, despite successful viral suppression, immune function does not completely normalize. Functional defects of myeloid cells are well-described during untreated disease [64, 65]. Our findings suggest that defects in monocyte function might exist in HIV+ individuals despite long-term ART treatment. Indeed, in addition to inflammatory SNAEs, HIV-infected individuals have persistently increased susceptibility to infections such as tuberculosis [4], and to multiple virus-associated cancers [5, 6]. Our data raise the possibility that myeloid dysfunction after ART could contribute to the sustained risk of these infectious and neoplastic complications. While it is plausible that this defect reflects a persistent effect of HIV immune dysfunction, chronic inflammation itself also can induce immunosuppression [66], and several of the downregulated genes in our HIV/ART monocytes have been associated with inflammation-related immunosuppression [67]. Future studies will be needed to define the mechanisms responsible.

Of note, several previous studies have compared transcriptomic patterns in HIV-infected subjects not on ART to those observed in healthy controls, and described multiple dysregulated pathways [46, 68–71]. In contrast, only a few have addressed monocyte gene expression in ART-treated subjects, and our study is the first to our knowledge to examine it in people with long-term ART suppression. One prior report described transcriptomic analysis of 5 ART-suppressed individuals compared with 4 healthy controls (as well as 5 untreated subjects), and identified 76 DEGs, of which 45 were upregulated and 31 downregulated in HIV/ART compared with controls [71]. However, those subjects were on ART for a mean of only 17 months, versus > 7 years for our subjects. Another study examined monocytes from untreated individuals compared with healthy controls' monocytes, and the impact on a subset of genes over the first 9 months of ART initiation. That study reported a large number of genes initially dysregulated, about half of which remained dysregulated after 9 months of treatment [72]. Our results differ considerably from those shorter-term ART studies, in that downregulation was the main direction of perturbation in HIV subjects compared with healthy controls. The distinct pattern in our study subject population is likely due to the duration of ART treatment, the relatively advanced stage at which ART was started, older subject age, or other factors. Furthermore, we also compared the gene expression patterns in these monocytes to a prior study of untreated subjects and found very little overlap in the dysregulated genes indicating that it is not simply a “less severe” reflection of that seen in untreated subjects. Taken together, these results suggest that long-term ART-treated individuals exhibit monocyte dysregulation that is qualitatively different from that observed in both untreated and short-term ART-suppressed individuals. Given the persistent susceptibility to certain infections and neoplastic complications, as noted above, this observation merits further investigation.

Our study has several strengths. Our subjects were selected for being at high risk of SNAEs, based on older age and beginning ART in advanced disease with relatively low CD4 counts. Furthermore, our subjects were on ART for > 7 years, which is highly relevant to the growing cohort of long-term treated people. Finally, our healthy controls were closely matched with our HIV/ART subjects based on age, race, gender, and smoking status. At the same time, our study has several limitations. While we studied a larger number of subjects than any prior HIV monocyte transcriptome studies, our stringent subject enrollment criteria resulted in a modest sample size, which limits statistical power. Also, within both the HIV and control groups, there was considerable heterogeneity, indicating multiple cofactors impacting surface markers, soluble factors, and gene expression. Monocyte purification (which here was ~95%) cannot completely eliminate any possibility of admixture with other cell types. While the consequences of surface markers such as TF and soluble factors such as CCL2 are strongly linked to SNAEs including neurological and cardiovascular disease in ART-treated individuals, future studies will be needed to identify both the mechanisms responsible for, and functional consequences of, dysregulated monocyte gene expression, as well as the potential impact of therapeutic targeting these pathways.

Finally, we selected participants on the basis of elevated CRP levels as an indicator of persistent inflammation, but it is likely that there are different patterns of residual immune activation, and use of other enrollment criteria (eg; TNF, sCD14, IL6, etc.) might have led to different findings. Combined with the heterogeneity we saw even within our stringently-selected participant group, it is likely that distinct “flavors of inflammation” exist that might not only differentially affect monocyte surface phenotypes, transcriptomic profiles, and soluble markers, but might even underlie distinct susceptibility to different comorbidities in ART-treated people living with HIV. Further research on the drivers and consequences of heterogeneity in residual inflammation in this population is warranted.

In conclusion, this study offers new insights into the perturbations in monocyte function in chronic HIV-1 infection on long-term ART. Our data reveal that monocyte subsets are largely normalized in these individuals, although with subtle abnormalities in levels of selected surface markers. We demonstrate elevated expression of a number of plasma biomarkers, particularly CCL2/MCP1, which is a key driver of neurological and cardiovascular SNAEs, and identification of a subset of subjects with coordinated increase among multiple mediators. Finally, we show global abnormalities of monocyte gene expression, dominated by downregulation of genes involved in various aspects of immune function. Taken together, our results shed light on the overlapping mechanisms that might contribute to both inflammatory complications of longstanding infection and, potentially, the overlapping issue of persistent innate immune dysfunction despite ART suppression.

## SUPPLEMENTARY MATERIALS

**Supplementary Table 1.** HIV+ viremic monocyte donors baseline characteristics.

Donor ID	Sex	Age	CD4 Count (cells/μL)	Viral Load (copies/ml)
1	F	48	352	22,170
2	M	46	458	23,262
3	M	40	300	10,611
4	M	54	353	24,075
5	F	46	332	29,587
6	F	55	306	3,283
7	M	36	504	61,470
8	M	35	201	56,643
9	F	35	1128	9,810
10	M	38	416	74,296
11	F	39	896	18,088
12	F	20	604	22,150
13	M	33	168	61,432

Gene expression data for HIV+ viremic individuals was obtained from [45, 46]. Table shows available clinical and demographic information.
